# Virology on the Internet: the time is right for a new journal

**DOI:** 10.1186/1743-422X-1-1

**Published:** 2004-08-26

**Authors:** Robert F Garry

**Affiliations:** 1 Department of Microbiology and Immunology Tulane University School of Medicine New Orleans, Louisiana USA

## Abstract

Virology Journal is an exclusively on-line, Open Access journal devoted to the presentation of high-quality original research concerning human, animal, plant, insect bacterial, and fungal viruses. Virology Journal will establish a strategic alternative to the traditional virology communication process.

## 

The outbreaks of SARS coronavirus and West Nile virus (WNV), and the troubling increase of poliovirus infections in Africa, are but a few recent examples of the unpredictable and ever-changing topography of the field of virology. Previously unknown viruses, such as the SARS coronavirus, may emerge at anytime, anywhere in the world. Viruses previously thought to be geographically restricted, such as WNV, may appear in new regions and spread rapidly. Poliovirus, once thought to be on the brink of elimination, has surged with a widespread distribution in nearly a dozen African nations that now poses a serious risk to the polio eradication initiative. Governments and individuals are increasingly aware of the threats posed by viruses, including established viruses, emerging viruses and the many viruses that are potential agents of bioterrorism. However, lack of information or misinformation regarding viruses can further exacerbate their impact on public health. There is an urgent need for a rapid forum for communications among virologists. Virology Journal will present high-quality original research concerning human, animal, plant, insect bacterial, and fungal viruses, while establishing a strategic alternative to the traditional virology communication process. Links to an extensive database of virology information on the Internet will be provided through our "All the Virology" (ATV) web site .

## Open Access

*Virology Journal*'s Open Access policy changes the way in which articles in virology can be published [1]. First, all articles are freely and universally accessible online as soon as they are published, so an author's work can be read by anyone at no cost. Second, the authors hold copyright for their work and grant anyone the right to reproduce and disseminate the article, provided that it is correctly cited and no errors are introduced. Third, a copy of the full text of each Open Access article is permanently archived in an online repository separate from the journal. *Virology Journal*'s articles are archived in PubMed Central [2], the US National Library of Medicine's full-text repository of life science literature, and also in repositories at the University of Potsdam [3] in Germany, at INIST [4] in France and in e-Depot [5], the National Library of the Netherlands' digital archive of all electronic publications.

Open Access has four broad benefits for science and the general public. First, authors are assured that their work is disseminated to the widest possible audience, given that there are no barriers to access their work. This is accentuated by the authors being free to reproduce and distribute their work, for example by placing it on their institution's website. It has been suggested that free online articles are more highly cited because of their easier availability [6]. Second, the information available to researchers will not be limited by their library's budget, and the widespread availability of articles will enhance literature searching [7]. Third, the results of publicly funded research will be accessible to all taxpayers and not just those with access to a library with a subscription. As such, Open Access could help to increase public interest in, and support of, research. Note that this public accessibility may become a legal requirement in the USA if the proposed Public Access to Science Act is made law [8]. Similar calls for a move to Open Access of all scientific research have been made recently by the UK government [9]. Fourth, a country's economy will not influence its scientists' ability to access articles because resource-poor countries (and institutions) will be able to read the same material as wealthier ones (although creating access to the Internet is another matter [10]). This is particularly relevant in virology as many viruses have regional, rather than global, distributions.

## Peer Review policy

Virology Journal will consider: research, book reports, case reports, commentaries, debate articles, hypotheses, methodology articles, reviews, short reports and short protocols. An editorial board of 30 members has been established [11]. In addition to these outstanding individuals, nine other distinguished virologists constitute an advisory board that will provide general oversight of the journal [11]. While initially all manuscripts will be submitted to my office, as Editor-in-Chief, as the volume of manuscripts increases, submissions in specific areas of virology (ie. large DNA viruses, plant viruses etc) will go directly to a Section Editor chosen by the author. The Editor-in-Chief or Section Editor will assign each research manuscript submitted to the journal to a member of the Editorial Board who will be known as the "monitoring editor". The monitoring editor will then appoint at least two ad hoc reviewers from experts in the field. Once the reviewers have provided their feedback, the monitoring editor makes the final recommendation. Managing Editor, David Sander will be available to assist authors with content and formatting issues not resolved during the review process. He will also assist the authors of review articles with integration of content with the ATV website (where appropriate). Articles will be published online immediately upon acceptance and soon after listed in PubMed.

## Competing interests

Critics of Open Access often suggest that Editors have a financial incentive to accept articles as more articles means more revenue. However, BioMed Central insists that decisions about a manuscript must be based on the quality of the work, not on whether the article-processing charge can be paid. This policy will certainly apply for *Virology Journal *whose authors and readers will benefit from learning about viruses in regions of the world with limited financial resources. No member of the editorial or advisory boards of *Virology Journal *or their Institutions will receive any portion of the article-processing charge.

It is also a BioMed Central policy that Editors should declare their competing interests. Several years ago, I suggested that it would be a useful policy for the Editors of scientific and medical journals to declare their competing interests on a yearly basis [12]. Few editors have accepted this suggestion, but by way of example I shall declare my own here:

"I declare that my institution holds or has applied for several United Stated and International patents based on technology developed in my laboratory. These patents or patent applications cover a range of technologies including diagnostic assays, human A-type retroviruses and a B-type retrovirus (betaretrovirus), and peptides that inhibit viral infectivity. Tulane University has licensed some of these technologies to private companies for commercial development (list available on request), and I receive royalties from these licenses. I have also served on several study sections for the National Institutes of Health and currently served as the Chair of a biodefense study section (SSS-Z). I receive a per diem and reimbursement from the NIH for service on the study sections. Except for mutual funds in a retirement account managed through Tulane University, I own no stocks or other commercial instruments."

## Conclusion

There are several outstanding virology journals covering all aspects of this dynamic field, but none of the general virology journals are exclusively published on-line or are Open Access. With the launch of *Virology Journal*, we hope to catalyse a fuller utilization of the Internet for scientific communication in virology drawing on our long experience with the ATV website. We welcome any advice and input.
